# Respiratory syncytial virus incidence and clinical outcomes in patients with inflammatory bowel disease: a National Veterans Affairs cohort study

**DOI:** 10.1093/ibd/izag103

**Published:** 2026-06-23

**Authors:** Nabeel Khan, Ramaswamy Sundararajan, Freddy Caldera, Francis A Farraye

**Affiliations:** Department of Gastroenterology, Corporal Michael J Crescenz VA Medical Center, Philadelphia, PA 19104, United States; Division of Gastroenterology, University of Pennsylvania, Perelman School of Medicine, Philadelphia, PA 19104, United States; Department of Gastroenterology, Corporal Michael J Crescenz VA Medical Center, Philadelphia, PA 19104, United States; Division of Gastroenterology and Hepatology, University of Wisconsin-Madison School of Medicine and Public Health, Madison, WI 53705, United States; Division of Gastroenterology and Hepatology, Mayo Clinic, Jacksonville, FL 32224, United States

**Keywords:** respiratory syncytial virus, inflammatory bowel disease, infection

## Abstract

**Background:**

Data describing the incidence and clinical outcomes of respiratory syncytial virus (RSV) infection among patients with inflammatory bowel disease (IBD) are limited.

**Methods:**

We conducted a nationwide retrospective cohort study using the Veterans Health Administration (VHA) database. Adults with IBD were identified using a previously validated algorithm and matched 1:2 to non-IBD controls using propensity score matching. Laboratory-confirmed RSV infections that occurred between January 1, 2021, and December 31, 2024, were identified using standardized laboratory codes and confirmed by manual chart adjudication. Primary outcomes included RSV incidence and hospitalizations. Secondary outcomes included intensive care unit (ICU) admission and patient outcomes stratified by IBD medication class.

**Results:**

After propensity score matching, 52 740 IBD patients and 104 997 non-IBD controls were included. Infection with RSV occurred in 219 patients with IBD and 426 non-IBD patients. Incidence rates of RSV were similar between cohorts across all years in our study period. Hospitalization related to RSV occurred in 18.26% of IBD-RSV patients compared with 13.15% of non-IBD patients (*P* = .08). ICU admission was rare in both groups (<1%). Among patients with IBD, RSV-related hospitalization did not differ significantly by medication class, when comparing biologic therapy (8.93%) with 5-aminosalicylic acid monotherapy (17.58%) (*P* = .15).

**Conclusions:**

In this nationwide cohort, patients with IBD, with predominantly lower exposure to advanced therapies, did not have higher rates of RSV infection or worse clinical outcomes compared with matched non-IBD controls. RSV outcomes were similar across IBD medication classes. These findings support current vaccination recommendations and provide reassurance regarding RSV risk among patients with IBD.

Key Messages *What is already known?*

There are limited data regarding the incidence and clinical outcomes of RSV infection among patients with IBD, particularly among those receiving immunomodulatory/immunosuppressive therapies.

*What is new here?*

In this nationwide cohort of patients with IBD, RSV infection rates and clinical outcomes were similar compared to those of matched non-IBD controls, with similar outcomes across various IBD medication classes.

*How can this study help patient care?*

These findings provide reassurance regarding RSV risk among patients with IBD and lower exposure to advanced therapies and support current vaccination recommendations for at-risk patients.

## Introduction

Inflammatory bowel disease (IBD), comprising ulcerative colitis (UC) and Crohn’s disease (CD), is an immune-mediated disorder treated with immunosuppressive/immunomodulatory drugs.^1–4^ As a result, patients with IBD have an increased risk of certain infections and these infections manifest themselves differently depending upon the severity of the disease and the concomitant use of medications.^5–7^ Although many infections have been studied extensively among the IBD population, there are limited data on respiratory syncytial virus (RSV) in this patient population.

RSV is a cause of acute respiratory illness in adults, with annual global estimates exceeding 1.5 million infections among older adults.^8^ This illness results in an estimated 123 000 to 193 000 annual RSV-related hospitalizations in the United States, with the highest rates among patients aged ≥65 years.^9^ The recent approval of the use of RSV vaccines in 2023 and 2024 in individuals aged ≥ 50 years supports the recognition of RSV morbidity among older adults.^10-12^ The American College of Gastroenterology (ACG) and American Gastroenterological Association (AGA) both recommend RSV vaccination for all patients aged ≥75 years and patients aged 50-74 years with risk factors for adverse RSV infection (ACG) and 60-74 years with similar risk factors (AGA).^13^^,^^14^

Very few studies have evaluated the prevalence and clinical course of RSV among patients with IBD, especially those exposed to immunomodulatory/immunosuppressive therapies. One study, from a multi-institutional database, demonstrated that adults with IBD who developed RSV were more likely to require hospitalization than non-IBD patients with RSV. The same study identified systemic corticosteroid exposure as a major risk factor for RSV-related hospitalization.^15^ However, these data were obtained through utilization of administrative coding to identify RSV infections and classify clinical outcomes without individual chart review.

To address the paucity of data describing the incidence and clinical outcomes of RSV infection among patients with IBD, we conducted a retrospective cohort study by leveraging a large national Veterans Health Administration (VHA) cohort.^16^ The objective of this study was to evaluate the incidence of RSV infection among patients with IBD compared with matched non-IBD controls in a large, nationwide cohort. Secondary aims included assessment of (1) hospitalization and ICU utilization following RSV infection, and (2) RSV outcomes stratified by IBD medication class.

## Methods

### Study design and participants

A retrospective cohort study was conducted using data from the VHA Corporate Data Warehouse (CDW), a centralized national repository that integrates clinical and administrative data from all Veterans Affairs Medical Centers (VAMCs) across the United States. We identified 2 cohorts: (1) an IBD cohort and (2) a non-IBD cohort. The IBD cohort included adult patients (aged ≥18 years) with a confirmed diagnosis of IBD determined by use of an algorithm that was previously validated.^17^ The study index date was January 1, 2021.

### Comparator group and matching

To assess the association between IBD and subsequent RSV infection, propensity score matching (PSM) was performed in a 1:2 ratio (IBD:non-IBD) using the PSMATCH procedure in the SAS Enterprise Guide (Version 8.3, SAS Institute Inc., Cary, NC, United States). Consistent with prior studies, matching variables included demographic characteristics (age at index, sex, race), comorbidities (diabetes mellitus, chronic lower respiratory disease, ischemic heart disease, heart failure, chronic kidney disease, solid organ transplant [liver or kidney], stem cell transplant, HIV), and behavioral risk factors (alcohol use disorder, smoking history, obesity).^7^^,^^15^^,^^18^

### Outcome ascertainment

The primary outcome was laboratory-confirmed RSV infection, identified through positive RSV test results recorded in the CDW between January 1, 2021, and December 31, 2024. Data for RSV testing were extracted from laboratory result tables using standardized LOINC (Logical Observation Identifier Names and Codes)codes (Table S1). This outcome was confirmed through manual adjudication of patient charts, and disposition of patients with RSV infection was listed as outpatient, emergency room (ER) visit, hospitalization, and intensive care unit (ICU) admission. The effects of exposure to IBD medication relative to RSV diagnosis were ascertained through manual chart review and defined as IBD therapy at the time of, or 30 days prior to, RSV diagnosis.

Secondary outcomes included the following: (1) clinical outcomes among the IBD-RSV cohort stratified by IBD medication exposure, (2) comparison of hospitalization among the IBD-RSV cohort among patients on 5-aminosalicylic acid (5-ASA) alone and patients on biologics with or without immunomodulators, (3) annual incidence of RSV during follow-up period. We also report vaccine utilization among these patients.

### Statistical analysis

Descriptive statistics were used to summarize relevant baseline demographic and clinical characteristics. Categorical variables were compared using the Chi-square test or Fisher’s exact test, based on expected cell counts. Incidence rates were calculated per 1000 person-years (PY) and incidence rate ratios (IRRs) with 95% CIs were computed to compare RSV infection rates between IBD and non-IBD cohorts across calendar years. A 2-sided *P* value < .05 was considered statistically significant. All data management and analyses were performed using R studio (R version 4.5.2).

## Results

### Baseline characteristics

After propensity score matching (PSM), the IBD cohort (*n* = 52 740) and the non-IBD cohort (*n* = 104 997) were well balanced across demographic and clinical variables. Most patients in both cohorts were male (88.5%) and White (77%-78%). Age distribution was similar, with approximately 47% of patients aged >65 years. Comorbidities such as diabetes mellitus (24%), obesity (13%), and chronic lower respiratory diseases (19%) were comparably distributed between groups (Table 1A). Among patients with IBD, most patients were exposed to 5-ASA (41.53%) followed by anti–tumor necrosis factor (TNF) therapies (12.64%) and thiopurines (6.25%) (Table S2; Table 1B).

**Table 1A:** Baseline characteristics of non-IBD and IBD patient cohorts.

Covariate		**Non-IBD, No. (%) (*n*** = **104** **997)**	**IBD, No. (%) (*n*** = **52** **740)**
**Sex**	F	12 115 (11.54%)	6066 (11.50%)
	M	92 882 (88.46%)	46 674 (88.50%)
**Age at index, y**	<50	27 071 (25.78%)	14 043 (26.63%)
	50-65	28 211 (26.87%)	14 134 (26.80%)
	>65	49 715 (47.35%)	24 563 (46.57%)
**Race**	Black	13 508 (12.87%)	6894 (13.07%)
	Hispanic	4920 (4.69%)	2622 (4.97%)
	Other	1833 (1.75%)	975 (1.85%)
	Unknown	2621 (2.50%)	1437 (2.72%)
	White	82 115 (78.21%)	40 812 (77.38%)
**Comorbidities, *n* (%)**			
**Diabetes mellitus**		25 920 (24.69%)	12 694 (24.07%)
**Obesity**		13 327 (12.69%)	6839 (12.97%)
**Smoking**		88 210 (84.01%)	43 857 (83.16%)
**Chronic lower respiratory disease**		20 319 (19.35%)	10 119 (19.19%)
**Ischemic heart disease**		18 853 (17.96%)	9368 (17.76%)
**Heart failure**		6092 (5.80%)	3266 (6.19%)
**Chronic kidney disease**		6988 (6.66%)	3754 (7.12%)
**Alcohol use disorder**		5508 (5.25%)	2898 (5.49%)
**HIV**		336 (0.32%)	222 (0.42%)
**Kidney transplant**		174 (0.17%)	123 (0.23%)
**Liver transplant**		162 (0.15%)	143 (0.27%)
**Stem cell transplant**		22 (0.02%)	18 (0.03%)

Abbreviations: IBD, inflammatory bowel disease.

**Table IB:** Medication exposure in the IBD cohort.

Medication	**Patients, No. (%) (*n*** = **52** **,740)**
**All medications**	52 740 (100%)
**5-ASA**	21 903 (41.53%)
**Immunomodulators**	4616 (8.75%)
**Biologics**	8767 (16.62%)
**No meds**	17 454 (33.09%)

Abbreviations: IBD, inflammatory bowel disease; 5-ASA, 5-aminosalicylic acid.

### Clinical outcomes

After manual adjudication through chart review, 219 IBD patients and 426 non-IBD patients had RSV during the follow-up period (Figure 1). Among patients diagnosed with RSV, hospitalization occurred in 18.26% (*n* = 48) of patients with IBD compared to 13.15% (*n* = 56) of non-IBD patients. However, this difference was not statistically significant (*P* = .08). Most patients were tested during emergency room visits, 65.30% of IBD patients and 67.37% of non-IBD patients. ICU admissions were rare (0.9% in both cohorts). Outpatient management occurred in approximately 16%-19% (*P* = .35) of both cohorts (Table 2).

**Figure 1:**
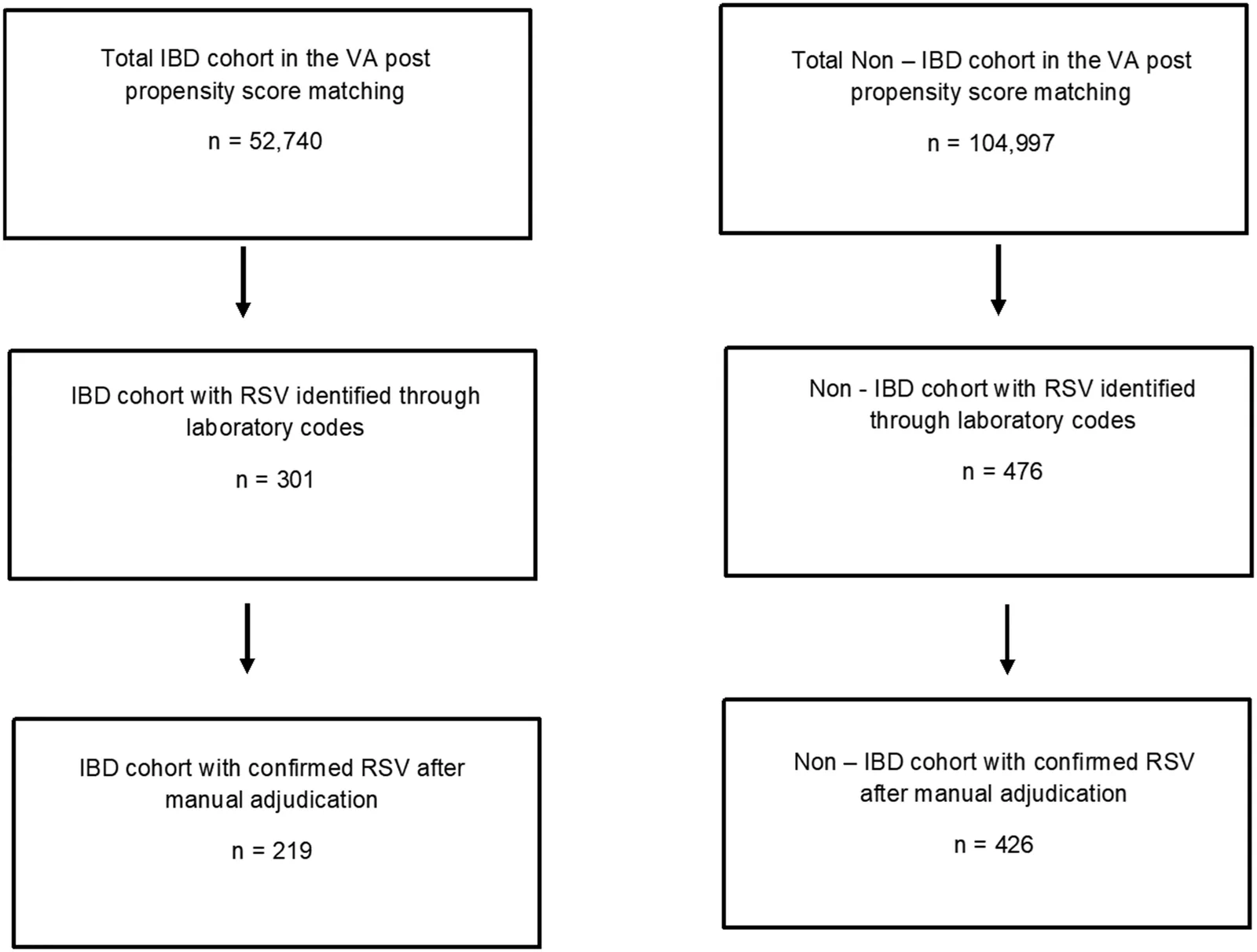
Cohort creation.

**Table 2:** Clinical outcomes between IBD-RSV and non–IBD-RSV cohort.

Outcomes	**IBD-RSV, No. (%) (*n*** = **219)**	**Non–IBD-RSV, No. (%) (*n*** = **426)**	*P* value
**Outpatient**	36 (16.44%)	83 (19.48%)	.35
**ER visit**	143 (65.30%)	287 (67.37%)	.60
**Hospitalization^a^**	40 (18.26%)	56 (13.15%)	.08
**ICU**	2 (0.91%)	4 (0.94%)	1.00

Abbreviations: ER, emergency room; IBD-RSV, inflammatory bowel disease/respiratory syncytial virus; ICU, intensive care unit.

a Hospitalization includes ICU.

### IBD medication exposure and outcomes by medication class

Among IBD-RSV patients (*n* = 219), 41.55% were on 5-ASA monotherapy, 21.0% were not on any IBD medications, and 25.57% were on biologics (with or without immunomodulators). The most frequently used biologics were adalimumab (7.31%) and infliximab (6.85%) (Table S3). Concomitant systemic steroid use, defined as steroid use at the time of RSV diagnosis or 1 month prior, was observed in 7.31% of patients (Table 3). Hospitalization rates varied by medication exposure (immunomodulators 30.0%, no IBD medications 28.3%, 5-ASA 17.6%, and biologics 8.9%). ICU admissions and mortality were rare across all treatment groups. Two patients required ICU admission, 1 patient in the concomitant steroid group and the other in the no-medication group. The patient in the no-medication group subsequently died (Table 4). No statistically significant difference was noted among RSV hospitalizations among patients with IBD on 5-ASA only compared to patients with IBD on biologics with or without immunomodulators (17.58% vs 8.93%, *P* = .15) (Table 5).

**Table 3:** IBD Medication exposure among the IBD-RSV cohort.

IBD medication exposure	**Patients, No. (%) (*n*** = **219)**
**No meds**	46 (21.00%)
**5-ASA alone**	91 (41.55%)
**Immunomodulators with or without 5-ASA**	10 (4.57%)
**Biologics ± immunomodulators**	56 (25.57%)
**Concomitant steroids**	16 (7.31%)

Abbreviations: IBD-RSV, inflammatory bowel disease/respiratory syncytial virus; 5-ASA, 5-aminosalicylic acid.

**Table 4:** Clinical outcomes by IBD medication exposure among IBD-RSV patients.^a^

Medication class	No. with RSV	Hospitalization^b^	ER visit	Outpatient visit	ICU admission	Death
**No meds^c^**	46	13 (28.26%)	24 (52.17%)	9 (19.57%)	1 (2.17%)	1 (2.17%)
**5-ASA alone**	91	16 (17.58%)	61 (67.03%)	14 (15.38%)	0 (0.00%)	0 (0.00%)
**Immunomodulators with or without 5-ASA**	10	3 (30.00%)	7 (70.00%)	0 (0.00%)	0 (0.00%)	0 (0.00%)
**Biologics ± immunomodulators**	56	5 (8.93%)	42 (75.00%)	9 (16.07%)	0 (0.00%)	0 (0.00%)
**Concomitant steroids**	16	3 (18.75%)	9 (56.25%)	4 (25.00%)	1 (6.25%)	0 (0.00%)

Abbreviations: ER, emergency room; IBD-RSV, inflammatory bowel disease/respiratory syncytial virus; ICU, intensive care unit; 5-ASA, 5-aminosalicylic acid.

a Values are No. (%) unless otherwise indicated.

b Hospitalization includes ICU.

c 1 patient was admitted in the ICU and died.

**Table 5:** RSV hospitalization among IBD patients on 5-ASA alone compared to IBD patients on biologics with or without immunomodulators.

Outcome	**Patients on 5-ASA diagnosed with RSV (*n*** = **91)**	**Patients on biologics with or without immunomodulators diagnosed with RSV (*n*** = **56)**	*P* value
**RSV Hospitalization**	16 (17.58%)	5 (8.93%)	.15

Abbreviations: IBD, inflammatory bowel disease; RSV, respiratory syncytial virus; 5-ASA, 5-aminosalicylic acid.

### Incidence of RSV over time

From 2021 to 2024, the incidence rate of RSV infection per 1000 patient-years (PY) remained relatively stable across both IBD and non-IBD populations. In 2024, the incidence was 1.30 per 1000 PYs in the IBD cohort and 1.21 per 1000 PY in the non-IBD cohort (incidence rate ratio [IRR], 1.07; 95% CI, 0.77-1.48; *P* = .688). No statistically significant differences in RSV incidence were observed between cohorts in any year analyzed (Table 6).

**Table 6:** Incidence rates of RSV among non-IBD and IBD patients during 2021-2024.

Year	Total IBD population	RSV cases	Incidence rate/1000 PY	Total non-IBD population	RSV cases	Incidence rate/1000 PY	Incidence rate ratio (95% CI)	*P* value
**2021**	52 743	38	0.72	105 000	81	0.77	0.93 (0.62-1.39)	.771
**2022**	50 655	65	1.28	100 723	116	1.15	1.11 (0.81-1.52)	.479
**2023**	48 736	55	1.13	96 831	116	1.20	0.94 (0.67-1.31)	.746
**2024**	47 003	61	1.30	93 338	113	1.21	1.07 (0.77-1.48)	.688

Abbreviations: IBD, inflammatory bowel disease; PY, person years: RSV, respiratory syncytial virus.

### Vaccine utilization

Among the IBD-RSV and non-IBD RSV population, 496 patients were aged  ≥ 50 years and eligible for the RSV vaccine per the US Food and Drug Administration (FDA). Of these patients, only 1 patient was vaccinated for RSV prior to RSV diagnosis.

## Discussion

In this retrospective, nationwide cohort of patients with IBD, we observed similar incidence rates of laboratory-confirmed RSV infection in patients with IBD when compared with matched non-IBD controls across 4 RSV seasons (2021-2024). These findings must be interpreted in the context of the medication exposure in our IBD cohort, in which a large proportion of patients were receiving no therapy or were on mesalamine, and therefore the findings may be most applicable to patients managed with less intensive treatment. When outcomes were stratified by IBD therapy, there was no difference among the patients exposed to different classes of medications, although the numbers of infections were low across the different classes of medications. Our study is, to our knowledge, the first to integrate manual chart adjudication of RSV clinical outcomes, allowing granular classification of outpatient management, emergency department visits, hospitalization, ICU admission, and death.

There is a paucity of data describing the incidence of RSV among patients with IBD. Smith et al., utilizing a large multi-institutional database, reported that among adults diagnosed with RSV, those with IBD were more likely to be hospitalized (adjusted odds ratio [aOR], 1.30; 95% CI, 1.06-1.59) and that recent systemic corticosteroid exposure nearly doubled hospitalization risk (aOR, 1.86; 95% CI, 1.30-2.59). ^15^ However, this study utilized administrative codes to identify outcomes. In contrast, using an actively followed IBD population with a non-IBD comparator and complete laboratory RSV capture through manual adjudication of patient charts, we observed that patients with IBD had hospitalization rates similar to those of patients without IBD. The majority of both IBD and non-IBD patients were diagnosed in the ER and did not require hospitalization. Moreover, the rates of ICU admission in both groups were exceedingly low at close to 1%. These data are very reassuring, in that patients with IBD do not suffer from a more severe form of RSV compared to non-IBD patients. Furthermore, patients with IBD do not have a higher risk of contracting RSV, with similar incidence rates between IBD and non-IBD patients over our study period. This finding suggests that even though patients with IBD may have underlying immune dysregulation, IBD itself and the medications used to treat it do not increase susceptibility to acquiring RSV infection. Overall, the rates were quite low and remained steady in each of the years of observation.

Within the limited number of patients in the IBD cohort who contracted RSV and were on biologic medications, there were no statistically significant differences between RSV hospitalization rates among patients with IBD on biologics with or without immunomodulator use compared to patients on 5-ASA therapy alone. This finding is consistent with findings reported by Smith et al., who did not observe any increased risk of RSV hospitalization among patients with IBD who were being treated with anti-TNF (aOR, 0.92; 95% CI, 0.52-1.61) and immunomodulator therapy (aOR, 1.40; 95% CI, 0.84-2.35) compared with patients on 5-ASA therapy.^15^ Because a large proportion of patients with IBD receive these immune mediating/suppressive medications, these findings provide insight into RSV-related outcomes in this patient population.

Despite FDA approval and the national availability of adult RSV vaccines during the study period, vaccination uptake in our cohort was strikingly low, with only 1 patient across both the IBD and non-IBD groups who developed RSV receiving the RSV vaccine. This finding must be interpreted in the context that RSV vaccines were approved late in our study period (May 2023), with clinical guidelines primarily recommending them initially for older adults aged  ≥ 75 years and individuals aged ≥ 50 years with specific risk factors.^13^^,^^14^^,^^19^ These guidelines limited the number of patients eligible for the vaccine during the observed period of our study. Large randomized controlled trials evaluating both protein-based as well as mRNA-based RSV vaccines demonstrated efficacy in preventing serious lower respiratory tract disease among older adults, with acceptable safety profiles.^10–12^ These findings are reassuring and support RSV vaccination in patients with IBD with existing age- and risk-based guideline recommendations, rather than being driven solely by IBD treatment type.

Our study has several notable strengths. First, it leverages data from the VHA, the largest integrated healthcare system in the United States, caring for more than 9 million veterans annually.^16^ Second, RSV diagnoses and clinical outcomes (outpatient visit, emergency department visit, hospitalization, or ICU admission) were verified through manual chart review, reducing the risk of misclassification that can occur when relying solely on administrative or ICD codes.^20^ Third, propensity score matching ensured an appropriate balance of key confounders known to influence RSV severity, including chronic obstructive pulmonary disease (COPD), chronic kidney disease, diabetes, and smoking history. Finally, the availability of detailed medication data enabled us to evaluate RSV outcomes across specific IBD treatment classes, providing insight into differential risk associated with various therapeutic strategies. However, our study is not without its limitations. The VA population predominantly consists of older male patients, which may limit the generalizability of the findings to younger or more sex-balanced and community-based IBD populations. At the same time, these characteristics were well balanced between the matched IBD and non-IBD cohorts, supporting the internal validity of the study comparisons. Medication exposure data were determined based on pharmacy dispensing records and may not accurately reflect patient adherence. However, our strict inclusion criteria, including documented VA prescriptions for IBD medications, likely mitigated this limitation. We acknowledge that due to the small number of RSV cases within each IBD medication subgroup, these analyses do not allow for definitive conclusions across treatment groups. Although 8767 patients in the overall IBD cohort were exposed to biologic therapies during the study period (Table 1B), the number of RSV events within these medication subgroups remained limited. Additionally, we did not evaluate disease activity, which may have resulted in residual confounding, because treatment groups may differ in disease severity.

In this nationwide cohort, patients with IBD did not have higher rates of RSV infection compared with matched controls, and RSV-related clinical outcomes did not differ significantly between the 2 groups. Additionally, similar clinical outcomes of RSV were noted between patients receiving biologics and those on 5-ASA therapies. However, due to the limited number of events, these findings should be interpreted cautiously. Pending additional studies, these findings support the age- and risk-based recommendations of the ACG and AGA for administering RSV vaccines for at-risk patients.

## Supplementary Material

izag103_Supplementary_Data

## Data Availability

The data for this manuscript cannot be made available in accordance with the HIPAA rules. However, de-identified data (without patient name and SSN), can be made available upon reasonable request.

## Author approval

All authors have reviewed and approved the final version of this article.
