# Supplementary material for: Respiratory syncytial virus incidence and clinical outcomes in patients with inflammatory bowel disease: a National Veterans Affairs cohort study
Source: Inflamm Bowel Dis. 2026 Jun 23;32(11):2103–9. doi: 10.1093/ibd/izag103 (PMC13722124; doi:10.1093/ibd/izag103)
Supplement: Supplementary file 1 [file izag103_supplementary_data.docx]

| **Description** | **LOINC code** |
| --- | --- |
| Respiratory syncytial virus A RNA [Presence] in Nasopharynx by NAA with probe detection | 77022-2 |
| Respiratory syncytial virus A RNA [Presence] in Specimen by NAA with probe detection | 30075-6 |
| Respiratory syncytial virus Ag [Presence] in Nasopharynx by Immunoassay | 68966-1 |
| Respiratory syncytial virus Ag [Presence] in Nasopharynx by Rapid immunoassay | 72885-7 |
| Respiratory syncytial virus Ag [Presence] in Nose | 33045-6 |
| Respiratory syncytial virus Ag [Presence] in Specimen | 31950-9 |
| Respiratory syncytial virus Ag [Presence] in Specimen by Immunoassay | 5876-8 |
| Respiratory syncytial virus Ag [Presence] in Specimen by Immunofluorescence | 5877-6 |
| Respiratory syncytial virus B RNA [Presence] in Nasopharynx by NAA with probe detection | 77023-0 |
| Respiratory syncytial virus B RNA [Presence] in Specimen by NAA with probe detection | 30076-4 |
| Respiratory syncytial virus RNA [Presence] in Lower respiratory specimen by NAA with non-probe detection | 92957-0 |
| Respiratory syncytial virus RNA [Presence] in Nasopharynx by NAA with non-probe detection | 82176-9 |
| Respiratory syncytial virus RNA [Presence] in Nasopharynx by NAA with probe detection | 76089-2 |
| Respiratory syncytial virus RNA [Presence] in Respiratory system specimen by NAA with probe detection | 92131-2 |
| Respiratory syncytial virus RNA [Presence] in Specimen by NAA with probe detection | 40988-8 |
| Respiratory syncytial virus RNA [Presence] in Upper respiratory specimen by NAA with probe detection | 85479-4 |

**Supplemental Table 1: Relevant laboratory diagnosis codes for RSV**

**Supplemental Table 2: Granular IBD medication exposure in the overall IBD cohort**

| **Medication** | **(n = 52,740)** |
| --- | --- |
| 5-ASA | 21,903 (41.53%) |
| Thiopurines | 3,298 (6.25%) |
| Anti-TNFs | 6,667 (12.64%) |
| Vedolizumab | 1,338 (2.54%) |
| Ustekinumab | 490 (0.93%) |
| Tofacitinib | 272 (0.52%) |
| Methotrexate | 1,318 (2.50%) |
| No meds | 17,454 (33.09%) |

**Supplemental Table 3: Granular biologic exposure in the IBD-RSV cohort**

| **IBD Medication Exposure** | **n = 219** |
| --- | --- |
| Adalimumab | 16 (7.31%) |
| Infliximab | 15 (6.85%) |
| Vedolizumab | 11 (5.02%) |
| Ustekinumab | 10 (4.57%) |
| Tofacitinib | 2 (0.91%) |
| Upadacitinib | 2 (0.91%) |
